# Automated Mapping of Land Cover Type within International Heterogenous Landscapes Using Sentinel-2 Imagery with Ancillary Geospatial Data

**DOI:** 10.3390/s24051587

**Published:** 2024-02-29

**Authors:** Kristofer Lasko, Francis D. O’Neill, Elena Sava

**Affiliations:** Geospatial Research Laboratory, Engineer Research and Development Center, 7701 Telegraph Road, Bldg 2592, Alexandria, VA 22315, USA; francis.d.oneill@erdc.dren.mil (F.D.O.); elena.sava@erdc.dren.mil (E.S.)

**Keywords:** land cover, land use, random forest, machine learning, arid, tropical, temperate

## Abstract

A near-global framework for automated training data generation and land cover classification using shallow machine learning with low-density time series imagery does not exist. This study presents a methodology to map nine-class, six-class, and five-class land cover using two dates (winter and non-winter) of a Sentinel-2 granule across seven international sites. The approach uses a series of spectral, textural, and distance decision functions combined with modified ancillary layers (such as global impervious surface and global tree cover) to create binary masks from which to generate a balanced set of training data applied to a random forest classifier. For the land cover masks, stepwise threshold adjustments were applied to reflectance, spectral index values, and Euclidean distance layers, with 62 combinations evaluated. Global (all seven scenes) and regional (arid, tropics, and temperate) adaptive thresholds were computed. An annual 95th and 5th percentile NDVI composite was used to provide temporal corrections to the decision functions, and these corrections were compared against the original model. The accuracy assessment found that the regional adaptive thresholds for both the two-date land cover and the temporally corrected land cover could accurately map land cover type within nine-class (68.4% vs. 73.1%), six-class (79.8% vs. 82.8%), and five-class (80.1% vs. 85.1%) schemes. Lastly, the five-class and six-class models were compared with a manually labeled deep learning model (Esri), where they performed with similar accuracies (five classes: Esri 80.0 ± 3.4%, region corrected 85.1 ± 2.9%). The results highlight not only performance in line with an intensive deep learning approach, but also that reasonably accurate models can be created without a full annual time series of imagery.

## 1. Introduction

Within a changing world, mapping and monitoring of land cover from remotely sensed imagery is a key characteristic of global change studies. Land cover plays a key role in many different environmental studies. At a broader scale, land cover is a key driver of climate change, with impacts on biogeochemical cycles, aerosols, and greenhouse gas emissions [1,2,3]. At regional and local scales, land cover and associated changes have many impacts. Land cover change has been linked with alterations to water pollution and water quality [4], including impacts leading to harmful algal blooms [5,6]. Forest cover loss has been linked to adverse effects on regional hydrometeorology [7]. Land cover change has been linked to land surface temperature changes, which is attributed to surface albedo effects [8,9,10,11]. Effects on soil erosion and sedimentation of water bodies have been related to land cover change [12,13]. Drought, fires, natural disasters, and the positive feedback from climate change have also played a role in driving land cover change [14,15,16]. The interconnection of land cover and land cover change demonstrates the importance of accurate methods to map land cover around the world.

Broad-scale land cover changes have been documented in various regions of the world. Logging, deforestation, reforestation, cropland expansion, and urban expansion have played a significant role in Southeast Asia [17], some of which have been driven by fires [18,19]. Therefore, monitoring and mapping of land cover is important for a variety of disciplinary studies in this region and beyond.

Efforts to map land cover at a global scale have been undertaken using a variety of datasets and methods. Early studies used 1 km or coarser resolution imagery with classification tree methods, a predecessor to the random forest. One such study yielded approximately 82% overall accuracy near-globally with 13 classes and used a variety of biophysical metrics as input features, such as NDVI amplitude and temperature at maximum NDVI [20]. A later study performed a similar assessment but leveraged decision trees [21]. By the mid-2000s, studies began producing global land cover maps using moderate–coarse-resolution MODIS 500 m or ENVISAT 300 m imagery. For example, the GLOBCOVER 2009 dataset was produced at a 300 m scale [22] with an accuracy of 73% [23], and annual global MODIS-based 500 m land cover products were generated based on supervised decision tree methods with manual training data using five of the prevalent classification schemes, including the IGBP global vegetation classification scheme and the University of Maryland classification scheme with a 75% overall accuracy [24]. These moderate–coarse-resolution datasets suffered from mixed pixel issues and variations in classification schemes, which made intercomparison difficult [25]. By the 2010s, with improved data storage and distributed processing, moderate resolution 30 m Landsat-based global products were released. For example, one study developed a global Landsat land cover map with eight land cover classes at an overall accuracy of 65%, using support vector machines (SVMs) and manually labeled training data [26]. The increased availability of cloud computing, using databases such as Google Earth Engine (GEE), led to more advancements. Recently, Sentinel-2 10 m and 20 m imagery, combined with deep learning and manual training data, has resulted in a global layer with about 85% accuracy for nine general land cover classes [27]. A recent study over Europe used time series Sentinel-2 imagery with automated data labels to produce a 13-class map with 86% accuracy [28]. The CORINE land cover mapping program has used computer-assisted photointerpretation to produce a series of 44 class land cover maps over Europe, with the most recent being the 100 m product based on Sentinel-2 imagery for the nominal period of 2018 [29,30]. The most recent product had an accuracy of about 92% [31]. Most of the mentioned studies leverage dense annual time series stacks of cloud-free satellite imagery.

Land cover datasets produced using multi-sensor fusion have emerged due to the increased capabilities of cloud computing and sensor data availability. One study produced annual datasets over a 30-year period using Landsat, MODIS, and AVHRR imagery with 80% accuracy [32]. The coarse imagery provided useful information at a near-daily scale about phenology, which led to an accurate outcome. Until recently, studies had not produced near-real-time land cover datasets, instead typically producing datasets annually, which made timely monitoring difficult. The Dynamic World dataset achieved near-real-time capability with an accuracy ranging from about 77% to 88%, depending on the validation method [33]. SAR, multispectral, elevation, and/or lidar imagery fusion combined with advanced deep neural network methods have also been applied in select regions, with overall accuracies ranging from 75% to 95% [34,35,36,37,38,39,40,41,42,43], including studies with automatically generated training data for self-supervised classification on select study sites [44,45]. The studies leveraging deep learning methods generally exceed the accuracy of shallow machine learning methods due to deep learning’s ability to better represent texture, morphology, and objects [46,47,48], but the accuracy can be poor when training data are of low quality or not geographically transferable [49,50,51]. Some studies, however, have had small-scale success in automating training data using region-growing and conditional random fields or other methods [52]. Lately, studies have combined pixel-based image segmentation, such as multi-layer perceptron, with patch-based convolutional neural networks to leverage the advantages of both methods [53].

Despite these advancements in deep learning methods, traditional shallow machine learning methods should not be overlooked due to their scalability and efficiency. A variety of studies have had success with shallow machine learning methods at country or region scales. One recent study evaluated the use of different temporal composites, which performed better than complete annual time series, and found that performance can be very high: overall accuracy was 89.8% [54]; similarly, temporal time series smoothing can yield about a 4% increase in accuracy [55]. Another study within the Brazilian tropical Savannah achieved 73% and 74% accuracy with random forest and support vector machines, respectively [56]. In several European countries, a random forest and support vector machine classifier resulted in Sentinel-2 scene accuracies ranging from 70% to 86% [57]. Other studies leveraging shallow machine learning with manual training labels have typically achieved accuracies in the range above 70% and below 90% [58,59,60,61] but with occasional higher accuracy exceptions [62,63]. The inclusion of texture and other spatial metrics has led to accuracies above 80% [64] or 90% [65], with automated training labels yielding 85% in one case [66]. It is important to note that such intercomparison of studies can be misleading, as accuracy metrics are typically impacted by the total number of classes, definition of classes, image resolution, local geography, and climate. Studies have evaluated deep-learning- and shallow-machine-learning-based classifiers and found the latter to have similar or slightly lower accuracy, typically with less than a 5% difference [52,67,68,69,70,71,72,73] but occasionally higher than 10% [74,75]. 

At higher resolutions (finer than 5 m), deep learning methods that take advantage of context, morphology, and texture are typically key for achieving strong accuracy [76,77,78,79,80]; however, shallow machine learning approaches operating with pixel-based methods [81] or object-based segmentation in conjunction with texture and ancillary data have achieved accuracies ranging from 70% to above 90% within local–regional study sites [82,83]. UAS imagery has also been classified using SVM or random forest combined with texture, shape properties, and other feature sets to produce accuracies above 85% [84,85,86]. Some of the previously mentioned studies are geographically limited, and the models may require further training and tedious manual data collection for broader application. To alleviate this issue of a lack of training data, models can be pretrained on a large ancillary dataset and then finetuned at the local scale with limited labels; furthermore, reference data can be generated based on center points derived from CenterNet and guided by transfer learning [87,88].

Currently, a variety of land cover datasets are available to users at moderate resolutions: Esri land cover [27], CORINE land cover [28,29], ESA World Cover [89], Dynamic World [33], Global Land Use Extent [90], and several others. Coarse-resolution datasets include Globcover [22], MODIS land cover [24], and several others. While these products are accurate, most are generated for fixed time periods using dense time series sets of images, which can be computationally demanding. If a user needs a current year map, it may not be available from these datasets in a timely manner. The Dynamic World dataset produces land cover based only on a single date, thereby lacking seasonal land cover. 

While previous studies have yielded accurate land cover classification schemes, many of these studies relied on dense time series stacks, manual or semi-supervised training labels, or computational deep learning methods. In this work, we propose a methodology that uses only two dates of imagery to capture some seasonal spectral variation, with automatically selected training data based on a combination of ancillary data, spectral, textural, and Euclidean distance characteristics. A random forest classifier was then fit to these training data for efficient processing on a local machine. The algorithm was optimized and evaluated on seven international Sentinel-2 granule-sized test sites of varied geographies and climates spanning five continents. After an intermediate set of land cover maps was generated along with more than 2800 manually labeled stratified random ground truth points, thresholds for automated training data labeling (spectral, textural, and distance) for each land cover type were evaluated across each scene using stepwise iteration to determine the optimal values per site, climate region (arid, temperate, and tropics), and across all sites combined (global). Several novel texture metrics were also included in the feature set, e.g., water texture. The resulting land cover model, based on two dates of imagery, was also evaluated against a temporally corrected model, which used an annual composite of NDVI 5th percentile and 95th percentile values to refine the model after classification. Different classification schemes were also computed and compared (nine-class, six-class, and five-class) against a deep-learning-based model hosted by Esri. Confusion matrixes were generated, and outputs were also compared by area and at the pixel level. Ultimately, this study offers novelty as it generates a near-globally compatible land cover algorithm that requires less than a full year of an imagery time series, can automatically generate training labels (no need for intensive manual labeling), and is efficient enough to be run on a local machine of average processing capability.

## 2. Study Area and Datasets

### 2.1. Study Area

The study sites were carefully selected to include areas representative of most of the geographies and landscapes across the world, with the exception of arctic, high latitude, and extreme mountainous regions. To best represent a land cover algorithm that would function at a near-global scale, we selected seven study sites that each span one Sentinel-2 granule size. These sites include arid, urban, tropical, temperate, rural, agricultural, and forested sites of varied terrain. Two arid sites, two tropical sites, and three temperate sites were selected. The study sites are described in Table 1. 

### 2.2. Sentinel-2 Imagery

Sentinel-2 Multispectral Instrument (MSI) imagery is provided by the European Space Agency [91]. We acquired the Bottom of Atmosphere (BoA) reflectance, Level 2A (L2A), from the Copernicus Open Access Hub. The L2A product contains geometrically and radiometrically corrected imagery, along with a quality assurance layer called the Scene Classification Layer (SCL) that contains information on clouds, cloud shadows, snow, water, and ice, which was used to mask the input imagery and obtain a snow mask. Sentinel-2 MSI imagery includes 13 spectral bands at three spatial resolutions (10 m, 20 m, and 60 m). We leveraged the 20 m dataset to take advantage of the two shortwave-infrared (SWIR) bands and red-edge bands, along with the RGB and NIR bands. For each site, a winter season and non-winter season image was acquired to capture spectral variation and seasonality. The processing algorithm for Sentinel-2 L2A imagery underwent changes for imagery acquired in late 2021 and thereafter. The most notable difference is that post-2021 L2A surface reflectance values have an offset value of 1000 added to them. Please note that all spectral band values reported in the methods have had this offset removed (where applicable), and reported values correspond to pre-late 2021 ranges. Table 1 shows a list of input imagery used for each site.

To improve classification accuracy around land–water interfaces, a water texture layer is computed using the SCL mask from both dates of Sentinel-2 imagery. For each date, a 5 × 5 pixel kernel is convolved over the entire image, taking the sum of pixels labeled as “water” by the SCL layer at each convolution step. This texture layer is then included as a training attribute when fitting the random forest classifier. This layer helps the classifier to delineate water boundaries and highlight variation in shorelines over time.

### 2.3. Landsat Global Manmade Impervious Surface (GMIS) Layer

The GMIS layer, provided for the year 2010 from 30 m Landsat imagery from the Global Land Survey, was downloaded from the Socioeconomic Data and Applications Center of NASA [92]. The distributed layer is composed of values ranging from 0 to 100, indicating the percentage of impervious surface for a given pixel. Using the Geospatial Data Abstraction Library (GDAL), we downscaled the GMIS layer to 20 m resolution using the nearest neighbor and ensured pixels were properly aligned between the Sentinel-2 imagery and this product [93]. Conversion from coarse to finer resolution yields some degree of inherent positional error but is unavoidable and would primarily apply to less common edge pixels. We included the GMIS layer in the logical decision functions for training data selection to increase the accuracy of our model, as described in the Methodology section.

### 2.4. Global Impervious Surface Area (GISA) Layer

The GISA layer was provided for the year 2016 using a full time series of imagery from Sentinel-2 at 10 m and Sentinel-1 SAR at 10 m resolution [94]. The dataset is a binary layer produced at a global scale with an overall accuracy of about 86%. It was generated from a previous 30 m Landsat-based layer [95] as well as training data from Open Street Maps and other sources combined with a complex set of spectral, spatial, temporal, and geometric rules. 

### 2.5. Global 2010 Tree Cover and Global Forest Loss Year

The 30 m Landsat-derived global 2010 tree cover layer contains a percentage of canopy closure for trees greater than 5 m in height [96]. We extracted all pixels greater than 10% canopy closure as tree cover pixels. Secondly, we used the global forest loss year product version 1.10 provided by the Global Land Analysis and Dynamics (GLAD) laboratory to refine the tree cover layer by removing any tree cover pixels with a forest cover loss year between 2010 and 2022. This 30 m layer was subsequently resampled, aligned, and reprojected to match the 20 m Sentinel-2 imagery for each of our study sites. The datasets are available here: https://glad.umd.edu/dataset (accessed on 13 October 2022).

### 2.6. Global Land Use/Land Cover (Esri)

The Sentinel-2 10 m land use/land cover product was provided on an annual basis for each year of 2017–2022. It was developed using a UNet convolutional neural network (CNN) architecture trained on manually labeled points across the world, resulting in nine land cover classes [27]. Because of discrepancies between land cover classes in this dataset and our study, we merged land cover classes to enable direct comparison (as described in the Methods section below). The dataset was resampled to 20 m pixels (majority reclassification scheme) to correspond with our Sentinel-2 20 m imagery. We acquired the year of imagery, which corresponded to the date of our Sentinel-2 imagery for each study area. In this study, we refer to this dataset as Esri land cover because it is freely available for download on their website (https://livingatlas.arcgis.com/landcover/, accessed on 20 October 2023); however, it was produced by Impact Observatory. 

### 2.7. Sentinel-2 Annual NDVI Percentile (5th and 95th) Composites

The harmonized Sentinel-2 surface reflectance product hosted on Google Earth Engine was used for evaluation of how the use of this dataset impacts land cover map accuracy via a series of post-classification corrections. This harmonized dataset enables compatibility between pre-2022 and post-2022 imagery when pre-processing methods were changed within the Sen2Cor processing system. The Sentinel-2 surface reflectance product was collected with less than 20% cloud coverage per scene. The Scene Classification Layer (SCL) was used to further mask out clouds, thin cirrus, and shadow pixels. The imagery was compiled for one year in conjunction with the corresponding year of data used for each study area. Subsequently, for each study site, one year of imagery was collected, corresponding with the original two-date images. This image set was used to compute the 5th percentile and the 95th percentile of NDVI. The 5th percentile is representative of local minima and is used instead of minimum NDVI as occasional cloud or shadow pixels would persist into the final output. The 95th percentile layer is used for temporal corrections as well.

## 3. Methods

The objective of this study is to devise a method to automatically generate training data and subsequently map land cover types at 20 m Sentinel-2 resolution. We tested across seven study sites representative of most global landscapes (except for arctic areas and extremely mountainous regions). The method should not need to manually generate training data labels. Furthermore, this study endeavors to create a method that maintains similar accuracy levels to deep-learning-based approaches that use large volumes of manually labeled training data. The third objective is to evaluate how accuracy varies when mapping land cover using two dates of Sentinel-2 imagery versus the same procedure but with the addition of an annual NDVI 5th and 95th percentile composite and a series of post-classification corrections based on these layers. The penultimate objective is to evaluate spatial variation and accuracy differences when generating nine-class land cover, six-class land cover, and five-class land cover products. Lastly, for comparison purposes, this study seeks to evaluate how the land cover models from this study compare with those produced by a deep-learning-based model using a full annual Sentinel-2 time series and manually labeled training points (Esri Redlands, CA USA Impact Observatory, Washington, DC, USA).

**Figure 1:**
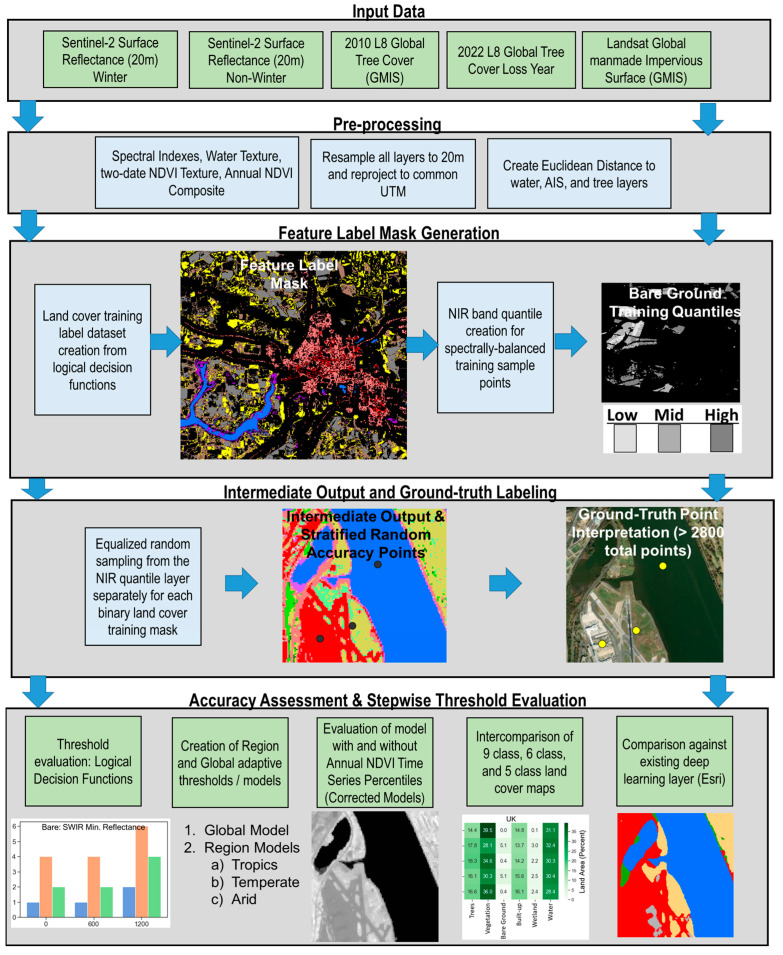
Flowchart of the general approach used in this study.

The general approach of this study is visualized in Figure 1. For each of the seven study sites, a Sentinel-2 surface reflectance image is acquired at 20 m spatial resolution during both winter and non-winter seasons, respective to each study site. Next, the ancillary datasets are processed, reprojected, and aligned to the Sentinel-2 datasets for each study area. The global tree cover extent layer is updated to 2022, and the global AIS dataset is both resampled to 20 m and resampled to binary bitmasks (forest/not-forest and AIS/not-AIS, respectively). The logical decisions used in this study are based on early decision tree approaches where entire images were classified using related functions [20,21]. In contrast, our approach uses these logical decision functions in conjunction with ancillary layers to sample only a portion of the image, which is then classified using a random forest. The image classification approach is loosely based on prior research from single land cover types (water, built-up) and expanded to a general nine-class land cover model [97,98]. Our logical decision function threshold values for water and AIS were taken from the two previously mentioned studies. We also introduce additional novelties in this study, such as temporal corrections with the 95th and 5th percentile annual NDVI layer and associated spatial and spectral decision functions, as well as comparison among six-class, five-class, and nine-class land cover schemes, which has not been thoroughly computed in the literature. The proceeding sections describe the specific steps used in this study.

A texture layer is generated based on the standard deviation of the difference of NDVI between the winter and non-winter imagery (Equation (1)) within a 3 × 3 pixel window. This texture layer is used during the creation of the binary AIS masks. The texture layer highlights areas of spatial variation in spectral signatures that are common in urban areas (e.g., different building materials, shadows, and mixed pixels) and is typically less common in areas confused with AIS, such as bare ground, where the spectral signature tends to be more consistent in neighboring pixels, and through time.

(1) $$texture_{ndvi}=SD_{kernel=3}\left(\left|ndvi_{non-winter}-ndvi_{winter}\right|\right)$$

### 3.1. Training Dataset Creation and Intermediate Output Land Cover Maps

The next steps focus on creating binary masks for each of the land cover types of interest. In this case, we initially generate a nine-class land cover product consisting of the classes shown in Table 2. 

The binary masks for each land cover type are intended to detect optimal cases (i.e., central examples) of each land cover class. Euclidean distance layers are generated for the GISA AIS layer, global forest cover extent layer, and the SCL water mask provided with the Sentinel-2 imagery (using both the winter and non-winter layers). The binary masks for each of the nine land covers are then generated based on logical decision functions using these data layers and spectral values. The logical decision functions were set with thresholds developed through experimentation over each study site and via findings from previous studies [97,98,99,100,101]. They were used to generate intermediate land cover layers for the creation of sample points, which are then used for threshold optimization and the creation of the final land cover maps. The proceeding intermediate land cover masks are created for the purpose of automatic training data selection within each site and are described in Table 3:

1. deciduous trees: 0.60 ≥ winter NDVI ≥ 0.25 AND non-winter NDVI min ≥ 0.6 AND B11 ≤ 2000 AND AIS distance ≥ 200 m (to avoid mixed pixels) AND Global Tree Layer Percent ≥ 10
2. evergreen trees: (winter NDVI min ≥ 0.65 AND B11 ≤ 1600 AND AIS distance ≥ 200 m) AND (non-winter NDVI min ≥ 0.60 AND AIS distance ≥ 200 m), AND Global Tree Layer Percent ≥ 10
  - evergreen trees: if latitude is between −10 and 10 degrees then deciduous trees are changed to evergreen.
3. vegetation (low NDVI): ((0.60 ≥ winter NDVI ≥ 0.30 AND B11 ≥ 1200) AND (0.60 ≥ Winter NDVI ≥ 0.4 AND B11 ≥ 1200)) AND treeCover distance ≥ 30 m AND AIS distance ≥ 200 m,
4. vegetation (high NDVI): ((non-winter NDVI ≥ 0.60) AND (Winter NDVI ≥ 0.20)) AND AIS distance ≥ 200 m AND treeCover distance > 100 m,
5. bare ground: (−0.1 ≤ NDVI AND winter NDVI ≤ 0.38 AND non-winter NDVI ≤ 0.39) AND AIS distance > 600 m AND B11 > 600,
  - bare ground (beach): an additional mask for bare ground adjacent to water. It uses the same decision logic as bare ground, but the Euclidean distance must be less than 50 m from SCL water. This mask gets combined with bare ground after training data generation.
6. water: ((AWEI ≥ −0.03 AND NDWI ≥ −0.03 AND MNDWI ≥ −0.03) AND B11 < 1000) for both winter and non-winter imagery [98], if only one image date meets this, then it is ephemeral water.
7. Wetland: (0.1 ≤ Winter NDVI ≤ 0.65) AND (0.1 ≤ Non-winter NDVI ≤ 0.60) AND (−0.20 ≤ MNDWI both dates ≤ 0.60) AND AIS distance ≥ 200 m AND distance from SCL water ≤ 100 m.
8. AIS (high density): (−0.5 ≤ Winter NDVI ≤ 0.35) AND (−0.5 ≤ Non-winter NDVI ≤ 0.60) AND (NDVI difference texture ≥ 3.5) AND GMIS AIS Percent Pixel Cover > 50%,
9. AIS (low density): (−0.5 ≤ Winter NDVI ≤ 0.5) AND (−0.5 ≤ Non-winter NDVI ≤ 0.75) AND (NDVI difference texture ≥ 3.5) AND (10% ≤ GMIS AIS Percent Pixel Cover < 50%).

To account for the effects of seasonal snow cover, the Sentinel-2 SCL snow mask is used. If a pixel is flagged as snow in the winter image, then only the non-winter image criteria need to be met from the logical decision functions to create the land cover masks [61]. There were no scenes with snow cover in both winter and non-winter dates.

After the nine binary land cover masks are created, they are subsequently used to generate training sample points for the image classifier. Specifically, the non-water masks are each stratified into 3 quantiles (using Jenks natural breaks) based on the NIR band value [102], while the water mask is stratified based on the NDWI. These quantiles are then used to generate equalized random sampling points to enable spectrally balanced training data. After stratification, 800 training points are generated for each land cover type. The beach bare ground is combined with the bare ground mask, and ephemeral water is combined with the wetland mask. Two training datasets are created for each mask: one “no-snow” and one “snow”. For the “no snow” training data, both the non-winter and winter criteria must be met, whereas for the “snow” set, the non-winter criteria must be met, and the winter scene must be labeled as snow by the SCL layer. Previous work has shown that classifier accuracy is not substantially affected by additional training points after a minimum training point count is achieved [103].

The training data samples, two dates of imagery, NDVI texture, water texture, and spectral indexes (NDVI, NDWI, AWEI, and MNDWI) are used to fit a random forest classifier containing the following parameters: 500 trees, max_depth = 30, max_samples = 500 as implemented in scikit-learn [104,105]. 

### 3.2. Accuracy Assessment and Stepwise Threshold Adjustment 

After the intermediate set of land cover maps was created, an accuracy assessment was conducted across each of the seven study sites. Pixels from the nine classes are selected via stratified random sampling for a total of 2858 points across the seven scenes (408 average points per scene) with a minimum of 30 points (when possible) per class to prevent under-sampling [100]. However, in one or two sites, we were unable to obtain 30 bare ground points more than 300 m apart from each other. We inferred estimated standard errors for each class, which typically resulted in class-specific standard errors of 0.05 to 0.15 depending on the scene; this information was then used with binomial probability sampling to select the total number of points per scene [106,107]. A minimum distance of 300 m between points was specified to reduce spatial bias [108]. The ground truth for each point was interpreted using a combination of high-resolution Worldview imagery, the original Sentinel-2 imagery, and spectral indexes. The overall accuracies, user’s accuracies, producer’s accuracies, and F1 scores are reported, as well as confusion matrixes for comparison purposes [109]. Confidence intervals of 95% uncertainty for the overall accuracies are computed for comparison purposes following best practice recommendations in accuracy assessment [106].

After the ground truth interpretation, the points are then used for the stepwise determination of optimal thresholds for each binary land cover mask. This is done because the initial thresholds used in the intermediate maps were based on exploratory analysis only and could be improved upon. The following variables were evaluated: bare ground AIS minimum distance (0 m, 300 m, 600 m), bare ground Band 11 minimum reflectance (0, 600, 1200), vegetation (low NDVI) NDVI minimum (0.3, 0.4) and NDVI maximum (0.5, 0.6), vegetation (low NDVI) Band 11 minimum (600, 1200), vegetation (high NDVI) AIS minimum distance (0 m, 100 m, 200 m) and NDVI minimum (0.1, 0.2, 0.3), water minimum spectral index (AWEI, NDWI, MNDWI) value (−0.1, −0.05, −0.03, 0) and maximum Band 11 reflectance (none, 1000, 1500), and wetland AIS minimum distance (0 m, 200 m) and MNDWI minimum (−0.25, −0.20, −0.15) and NDVI minimum (−0.05, 0.0, 0.1). An additional evaluation was performed where natural breaks (Jenks) separation was used as an adaptive threshold to differentiate several classes. Specifically, deciduous and evergreen trees, AIS (low density) and AIS (high density), and vegetation (low NDVI) and vegetation (high NDVI). Note that for AIS (low density) and AIS (high density), prior research found GISA to be slightly more accurate than GMIS [97], and we used those as the final thresholds in our study with AIS (high density): winter NDVI max (0.15), non-winter NDVI max (0.25); AIS (low density): winter minimum NDVI (0.15), non-winter minimum NDVI (0.25), winter maximum NDVI (0.65), non-winter maximum NDVI (0.45) [97].

### 3.3. Model Correction Using Annual NDVI Time Series Statistics

The annual Sentinel-2 NDVI composites were generated to include the 95th and 5th percentile values per pixel within all imagery with less than 15% cloud cover for the year corresponding to the original two-date Sentinel-2 imagery for each study site. Further cloud masking was conducted using the SCL cloud, thin cirrus, and shadow masks. The 95th and 5th percentile values were used in lieu of a minimum and maximum to avoid false values due to clouds and shadows that are not detected by the SCL mask. These layers can be used to detect drastic spectral changes to a pixel that are missed by the two-date classification output. This model correction is built upon early logical decision tree-based land cover studies [20,21].

Using the 95th and 5th percentile NDVI layers together with the GISA AIS layer, the following modifications are made to the land cover model and are then compared against the unmodified model and the ground truth points:

1. The first correction is the conversion of water pixels from the two-date Sentinel-2 classification into wetland/ephemeral water pixels if the NDVI_95th_percentile value > 0.60.
2. The second correction converts vegetation (low NDVI or high NDVI) or bare ground pixels into wetland/ephemeral water pixels if the NDVI_05th_percentile value < −0.15.
3. The third correction converts bare ground pixels into vegetation (low NDVI) if the NDVI_95th_percentile value is between 0.5 and 0.7 or vegetation (high NDVI) if the NDVI_95th_percentile value is greater than 0.7.
4. The fourth modification converts likely false positive AIS pixels into vegetation (low NDVI or high NDVI) or bare ground based on the 2nd or 3rd correction if the AIS pixel is greater than 6 km from an AIS pixel in the GISA AIS layer.
5. Probable false wetland pixels are modified to vegetation (low NDVI or High NDVI) or bare based on the 2nd or 3rd modification function if the pixel is greater than 5 km from a water pixel. Probable false positive wetland pixels occurring in urban areas are converted to AIS pixels if they coincide directly with a GISA AIS pixel and the NDVI_95th_percentile value is less than 0.7.
6. Another modification changes evergreen tree pixels to deciduous tree pixels if the NDVI_5th_percentile value is less than 0.4. If a deciduous or evergreen tree pixel NDVI_5th_percentile value is less than 0.2, then it is converted to vegetation (high NDVI or low NDVI based on the original decision function).
7. Lastly, a decision function is created to modify probable false positive AIS (low density and high density) pixels to bare ground based on how large clusters of AIS pixels in urban areas tend to have heterogeneous spectral signatures and often include a mix of vegetation, trees, or other mixed pixels that increase the NDVI above a nominal level. Comparatively, large swaths of bare ground found in arid regions will tend to have consistently low NDVI values through time. This modification changes an AIS (low density of high density) pixel to the bare ground if the maximum NDVI_95th_percentile value within a 55 × 55 pixel window (~1.1 km × 1.1 km) does not exceed 0.3.

These modifications are evaluated in the accuracy assessment and compared against the unmodified models.

### 3.4. Adaptive Regional Thresholds and Intercomparison

After threshold evaluation was completed, optimal thresholds were selected for each of the three regional threshold groups: temperate region (U.K., U.S., and FR imagery), tropics region (IN and BR imagery), and arid region (EG and QA imagery). The thresholds were also evaluated and compared globally (optimization across all seven scenes). 

The nine-class land cover dataset was condensed into six-class and five-class datasets for evaluation and comparison purposes. The specific classes are shown in Table 1, where the six-class and five-class models combine similar classes for the primary purpose of comparing against the Esri/Impact Observatory land cover dataset due to differences in land cover class definitions. The eight classes (and snow cover) produced in the Esri model do not align with the nine classes from our model, making direct comparison impossible. Thus, we combine the Esri classes of “rangeland” and “crops” into “vegetation”. The remaining classes are generally suitable for comparison with the six-class condensed model from this study. It is important to point out that the Esri land cover classes are defined slightly differently than in our study, and these differences will be analyzed and explored in the Results and Discussion sections.

## 4. Results

After the intermediate land cover maps were generated from the initial set of thresholds, ground truth points were created and interpreted on each of the seven sites and used to evaluate the stepwise threshold changes described in the Methods section.

### 4.1. Stepwise Thresholds of Land Cover Masks

**Figure 2:**
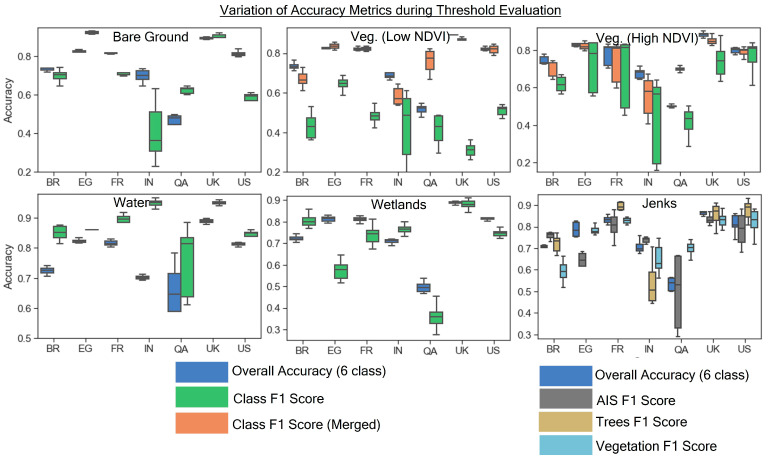
Visualization of how the overall accuracy and F1 scores varied during stepwise threshold evaluation illustrated by each study site and each binary land cover mask that is subsequently used for training data selection. The right-side legend applies only to the “Jenks Separation” sub-figure. The left-side legend applies to the remaining 5 sub-figures.

To measure changes in model accuracy, stepwise threshold adjustments were used to evaluate 15 different decision functions for the binary land cover masks. Figure 2 illustrates how the accuracy metrics vary when the thresholds are changed for each binary land cover mask. Several notable variations were observed: bare ground class F1 score for India had high levels of variation across bare ground, as well as both vegetation layers. The QA scene had notably high variation in water class F1 score and overall accuracy resulting from the different water thresholds, with overall accuracy ranging from about 0.6 to 0.78. The highest observed variation in accuracy for all scenes was the vegetation (high NDVI) class, where most scenes had substantial variation in the class F1 score, overall accuracy, and merged (low NDVI and high NDVI vegetation classes combined) F1 score. A major contributor to this effect was probably the fact that vegetation (high NDVI) occupied a substantial proportion of land cover for many of the scenes. Evaluation of the natural breaks (Jenks) threshold set to “true” or “false” for separating vegetation (low NDVI and high NDVI), trees (deciduous and evergreen), and AIS (low density and high density) resulted in notable variation in accuracy metrics especially with the AIS threshold in the QA scene. The main finding from this analysis shows that threshold variation for vegetation and the Jenks method generally yielded substantial changes in accuracy for most sites. Meanwhile, for the other classes, threshold variation was less pronounced (bare ground, wetlands, water), and substantial accuracy variation was limited to select sites (i.e., QA for water, IN for bare ground).

**Figure 3:**
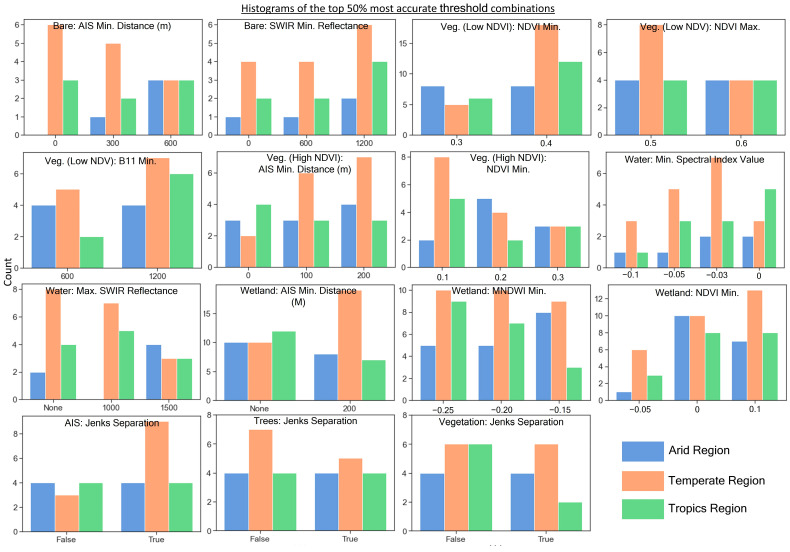
Bin plots of the top 50% most accurate stepwise threshold combinations for the 15 different variables evaluated. Bins are grouped by region to highlight trends.

Figure 3 shows histogram plots of thresholds from the top 50% of the most accurate combinations evaluated. This plot is separated into the respective regions (arid, temperate, and tropics), and it highlights several observed trends. For the bare ground mask, a 0 m minimum distance from the GISA AIS layer threshold was the most common result for the temperate scenes, whereas arid scenes performed best with the 300 m and 600 m distance thresholds. This is likely due to the combination of spectral similarity and high bare ground presence common among the two arid scenes. For the AIS natural breaks (Jenks) separation, there was no apparent impact for the arid and tropics regions, whereas enacting the natural breaks separation was common for the most accurate threshold combinations in the temperate scenes. For the wetland mask, the temperate region appeared to benefit the most from the 200 m minimum GISA AIS layer distance from wetland training pixels, whereas the trend was not clear in the other regions. This is likely because the wetland areas are very close to urban areas in the temperate scenes but less so in the other regions.

Based on evaluation against the 2858 accuracy assessment points (spread across the seven sites), the optimal threshold values were determined for each binary land cover mask (the training masks used for generating the random sample points for the random forest classifier). These thresholds were optimized by region (arid, temperate, and tropics) and globally (all seven sites combined) and are shown in Table 4. Use of the natural breaks (Jenks) method to separate AIS (low density) and AIS (high density) resulted in higher accuracy (temperate region) and minimal difference (arid, tropics), and separation of vegetation (high NDVI) and vegetation (low NDVI) using natural breaks (Jenks) resulted in minimal difference (arid, temperate) and reduced accuracy (tropics). The use of natural breaks (Jenks) to separate deciduous trees and evergreen trees resulted in minimal discernable differences across all regions.

### 4.2. Land Cover Maps with Optimized Thresholds and Temporal Corrections

**Figure 4:**
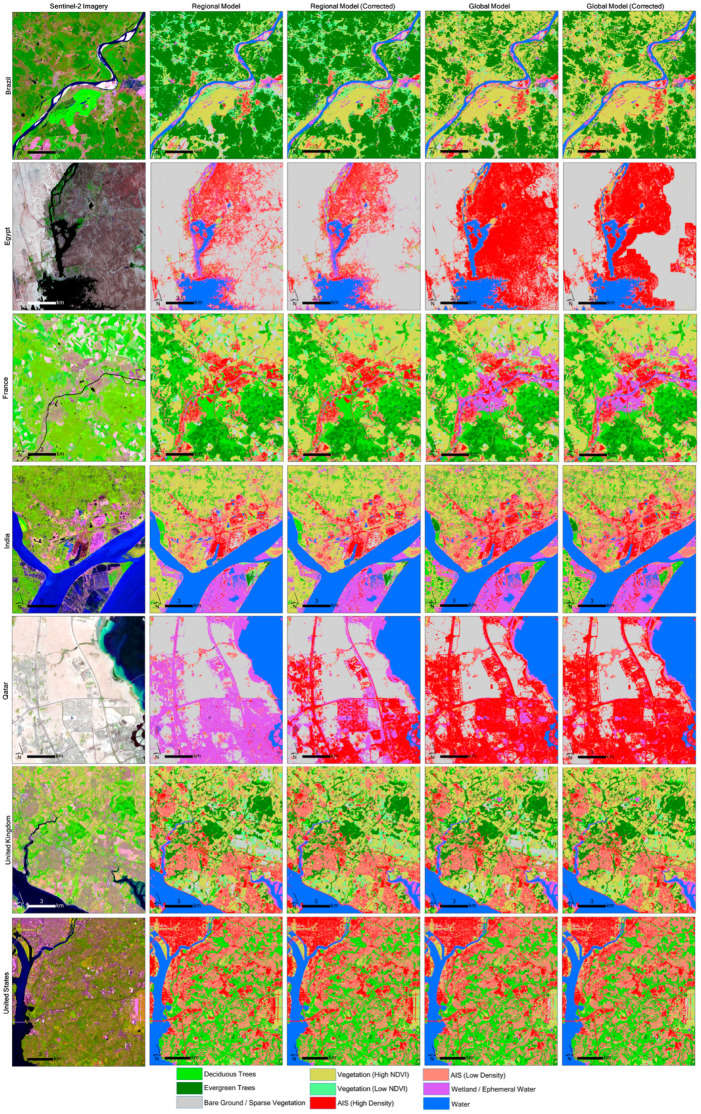
Sentinel-2 imagery with the region, region-corrected, global, and global-corrected models shown for selected areas of variation within each study site. A high-resolution version of this figure is also available.

**Figure 5:**
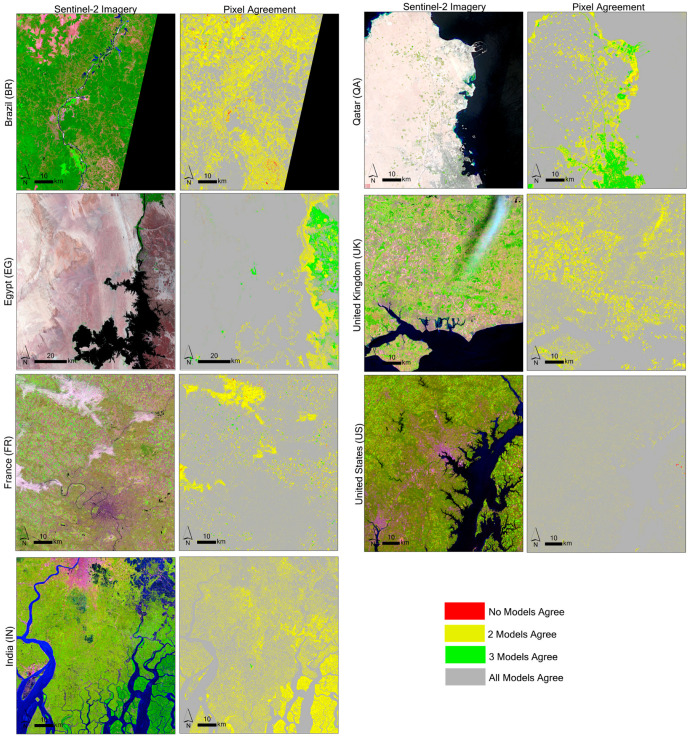
Pixel agreement maps highlighting pixel agreement of land cover type for the region, region-corrected, global, and global-corrected nine-class models.

The nine-class land cover model was generated for the seven study sites based on the regional models and global models for each site, as shown in Figure 4. The initial and corrected land cover outputs are shown for comparison and are zoomed over areas with variation for each study site. The BR site illustrates areas detected as bare ground in the regional and global models, which are modified to vegetation (high NDVI) in the corrected model due to the NDVI_95th_percentile layer, indicating the presence of vegetation in those pixels. Noticeable difference in evergreen tree presence between the global and regional models is also observed in this site, with the global model appearing to incorrectly label some pixels as vegetation (high NDVI). Within the EG site, the maps show AIS commission errors prominent along the edges of water bodies, as well as in hilly terrain on the East side of the image for both the global and regional models. Within the FR site, two notable patterns are observed: (1) groups of wetland pixels are detected over the urban area in both global models due to clouds in the winter image; however, this pattern does not appear in the regional model, likely due to the improved thresholds. (2) The bare ground detected over croplands in the initial models was detected as vegetation in the corrected models because vegetation presence was observed in the NDVI_95th_percentile composite. A similar pattern can be seen in the U.K. site. The IN and QA sites show examples where the global model appears to outperform the regional models by better mapping AIS areas; this is likely due in part to natural variation in the training data selected due to the random sampling and errors in the GISA AIS extent layer along wetlands. Figure 5 illustrates the variation and pixel agreement between the four models across the entire study area at a broader scale.

### 4.3. Nine-, Six-, and Five-Class Model Accuracies and Comparison with Esri Model

**Figure 6:**
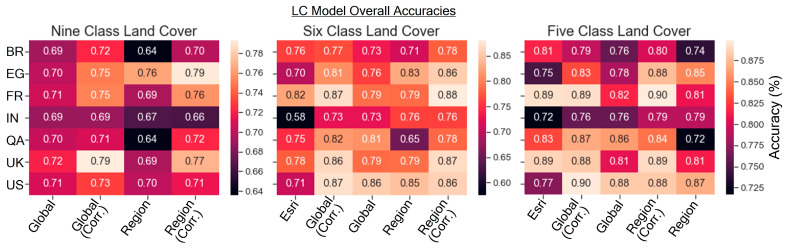
Overall accuracies for the nine-class, six-class, and five-class land cover schemes shown for the global and region models from this study and the Esri/Impact Observatory model for comparison.

The overall accuracies are shown in Figure 6 for the nine-class, six-class, and five-class land cover models. The six-class output was created by merging the two AIS classes and the two vegetation classes from the nine-class output; the wetland and water classes were additionally merged to create the five-class output to enable comparison with the Esri land cover model. Figure 6 illustrates that the nine-class model’s overall accuracies ranged from 0.66 to 0.79 by scene. The corrected models show about a 5% average increase in accuracy, with very high increases in several scenes, such as the FR regional model (0.69 to 0.76), the QA regional model (0.64 to 0.72), and the U.K. regional model (0.69 to 0.77). Accuracy was slightly reduced when correcting the IN site (0.67 vs. 0.66). For the six-class models, the overall accuracies ranged from 0.76 to 0.88 (region-corrected model), 0.73 to 0.87 (global-corrected model), with the global model yielding higher accuracy in some scenes and the uncorrected regional model for QA yielding very low accuracy for the uncorrected regional model (0.64), but much higher in the corrected model (0.72). The overall accuracy for the five-class models was 79.9% region, 85.1% region corrected, 81.0% global, and 84.6% global corrected. The accuracies for the six-class models were 76.85% region, 82.7% region corrected, 77.9% global, and 81.7% global corrected. The accuracies for the nine-class models were 68.4% region, 73.1% region corrected, 70.2% global, and 73.4% global corrected.

The overall accuracy of the six-class and five-class models was directly compared to the ESRI deep learning model, as shown in Figure 6. Across the six-class land cover schemes, the Esri model performed less accurately than the global and region-corrected models for all sites, with overall accuracies ranging from 0.58 to 0.82 by scene. The lowest accuracy was seen in the IN site and the highest in the FR site. The performance was closer under the five-class scheme, where the Esri model performed more accurately than the uncorrected region model in 4/7 sites (U.K., QA, FR, BR). The five-class Esri model has drastically higher overall accuracies in all seven scenes when compared to the six-class Esri model. This is attributed largely to errors between the water and wetlands classes, which are elided when those cover types are merged in the five-class model.

### 4.4. Class-Specific Accuracies

**Figure 7:**
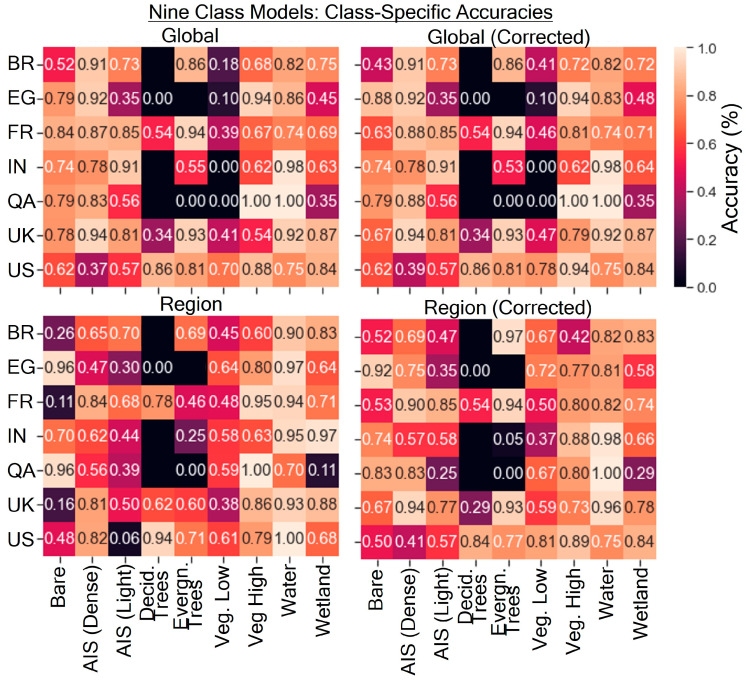
Class-specific accuracies for the four models from this study for the nine-class land cover scheme. Black, unannotated spaces indicate the absence of that land cover type in the scene.

The class-specific accuracies for the nine-class models are shown in Figure 7. Relatively higher changes in class-specific accuracy between the corrected and uncorrected models were seen in the bare ground, vegetation (low NDVI), and vegetation (high NDVI) classes. The region-corrected model generally performed more accurately than the global-corrected model in the two vegetation classes, which made up most of the land area in many of the scenes. However, some of the class-specific accuracies, such as AIS (low density and high density), were lower in the regional model than in the global model; these classes often made a relatively small proportion of most scenes. The QA and EG sites had class-specific accuracies of 0 for evergreen trees or deciduous trees or vegetation (low NDVI), as these classes were not detected in the models but did make up only a small fraction of the landscape. Within the IN site, minimal vegetation (low NDVI) pixels were present in the site and not detected by the models.

**Figure 8:**
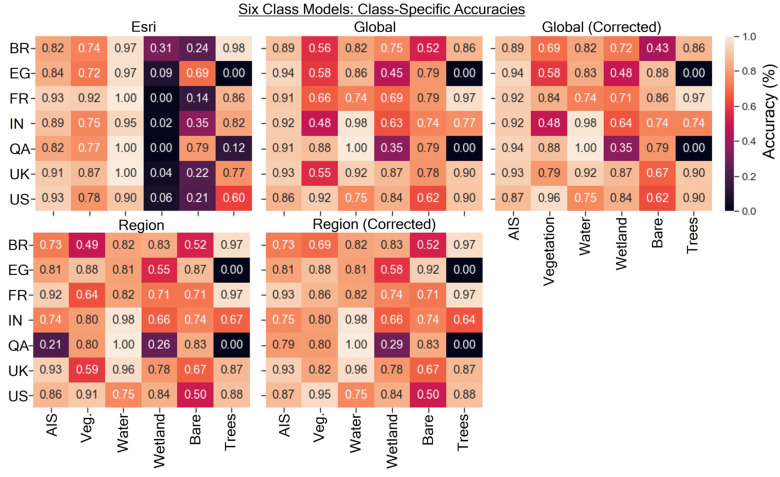
Class-specific accuracies for the four models from this study for the six-class land cover scheme. Black, unannotated spaces indicate the absence of that land cover type in the scene.

The class-specific accuracies for the six-class models are shown in Figure 8. The Esri model appears to have abysmally low accuracies in the wetlands and bare ground categories while typically exhibiting relatively higher accuracies in the water and AIS categories when compared to our study’s models. The Esri model exhibited class-specific accuracies above 0.95 for water in 6/7 scenes, with perfect accuracy in three scenes (U.K., QA, and FR). The global-corrected model generally yielded higher accuracies in the AIS category than the region-corrected model in most scenes, whereas the region-corrected model generally performed better in the vegetation category.

**Figure 9:**
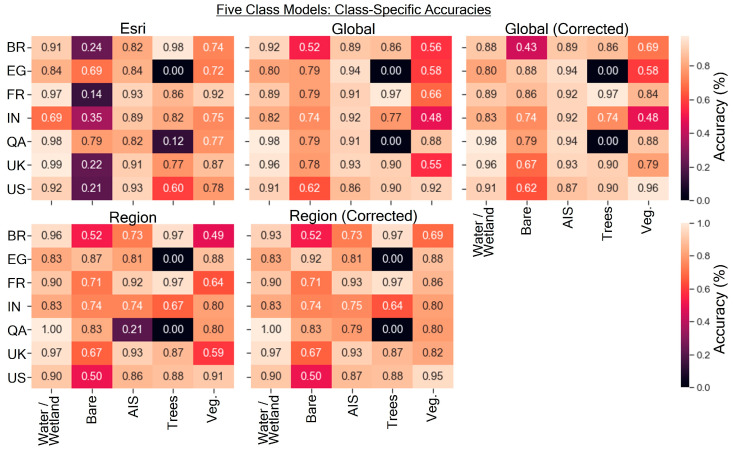
Class-specific accuracies for the four models from this study for the five-class land cover scheme. Black, unannotated spaces indicate the absence of that land cover type in the scene.

The class-specific accuracies for the five-class models are shown in Figure 9. The combined wetland/water class resulted in the Esri model exhibiting nearly comparable accuracy to the results of this study for that class, except for the IN site, where the Esri model struggled. Among the different classes, bare ground/sparsely vegetated typically had the lowest accuracy for both Esri and the models from this study. The vegetation class accuracy for the global and region-corrected models from this study exhibited the most drastic increases in accuracy when compared to the uncorrected models.

### 4.5. Confusion Matrix Comparison with Esri Model

The confusion matrixes for the five- and six-class models (regional models from this study and the Esri model) are shown in Table 5. The six-class region-corrected model had an overall accuracy of 82.8% (±3.1%) vs. the corresponding Esri model with 72.8% (±3.5%). For the five-class models, the region-corrected model was slightly more accurate than the six-class model at 85.1% (±2.9%), whereas the five-class Esri model had a drastic increase in accuracy over the corresponding six-class model, at 80.0% (±3.4%). Investigation of the confusion matrixes reveals several interesting patterns. For both six-class models, the wetlands category had the most omission errors (lowest producer’s accuracy). While the wetland omissions were balanced between trees, AIS, vegetation, and water for the region-corrected model, the Esri model had nearly all wetlands omission errors incorrectly mapped as water, with a sizable minority mapped as vegetation. This trend explains why the Esri model experiences a drastic increase in overall accuracy when converted into the five-class output. The second highest rate of omission errors was found in the bare ground category for both six-class models (producer’s accuracy of 62.4% and 81.9% for Esri and region-corrected model, respectively). Omitted bare ground pixels in the region-corrected model were mostly mapped as AIS pixels, likely due to the very similar spectral signatures. The Esri model, meanwhile, had relatively few bare ground pixels wrongly mapped as AIS, likely due to the contextual nature of the convolutional neural network. The majority of omitted bare ground pixels were incorrectly mapped as vegetation in the Esri model. Omission errors of trees in the Esri model were largely incorrectly mapped as AIS. Overall, for our study, wetland and bare ground had the highest omission errors, and AIS and wetland had the highest commission errors. Future improvements on these errors could yield drastic accuracy increases.

The confusion matrixes for the corrected nine-class global and region-corrected models are shown in Table 6. The results are interesting, with the region-corrected modeling yielding an overall accuracy of 73.2% (±1.9) and the global-corrected model with 73.4% (±1.8) with their 95% confidence intervals overlapping and indicating that they are not significantly different. This difference overall is primarily due to poor AIS accuracy in the QA scene for the region model, as shown in Section 4.4. For both models, we can observe the most confusion between the two AIS land covers, AIS and bare ground, and between high and low NDVI vegetation classes. Interestingly, the region-corrected model had much lower producer’s accuracy for both AIS categories than the global-corrected model. The region-corrected model had substantially high producer’s accuracy for the low NDVI vegetation category. The user’s accuracy was similar between the models, with more pronounced differences between evergreen trees, deciduous trees, and low NDVI vegetation.

### 4.6. Spatial Intercomparison of the Region-Corrected and Esri Models

**Figure 10:**
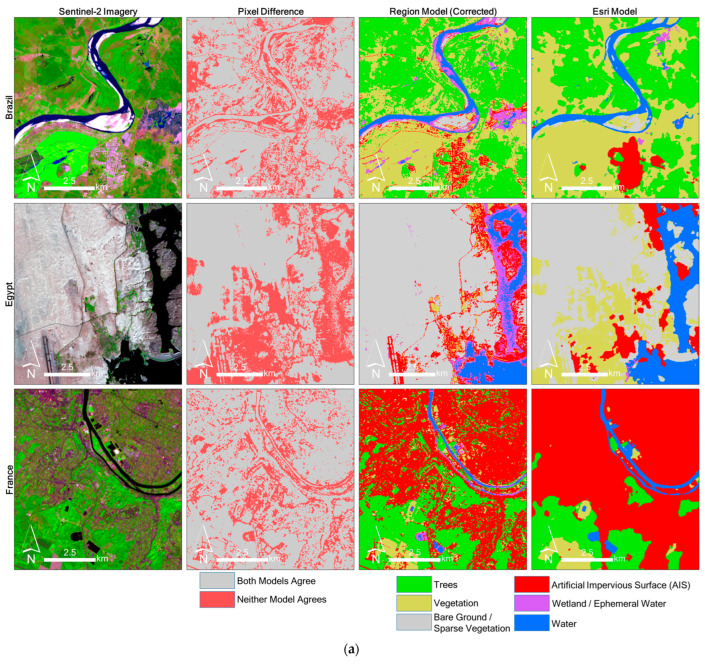

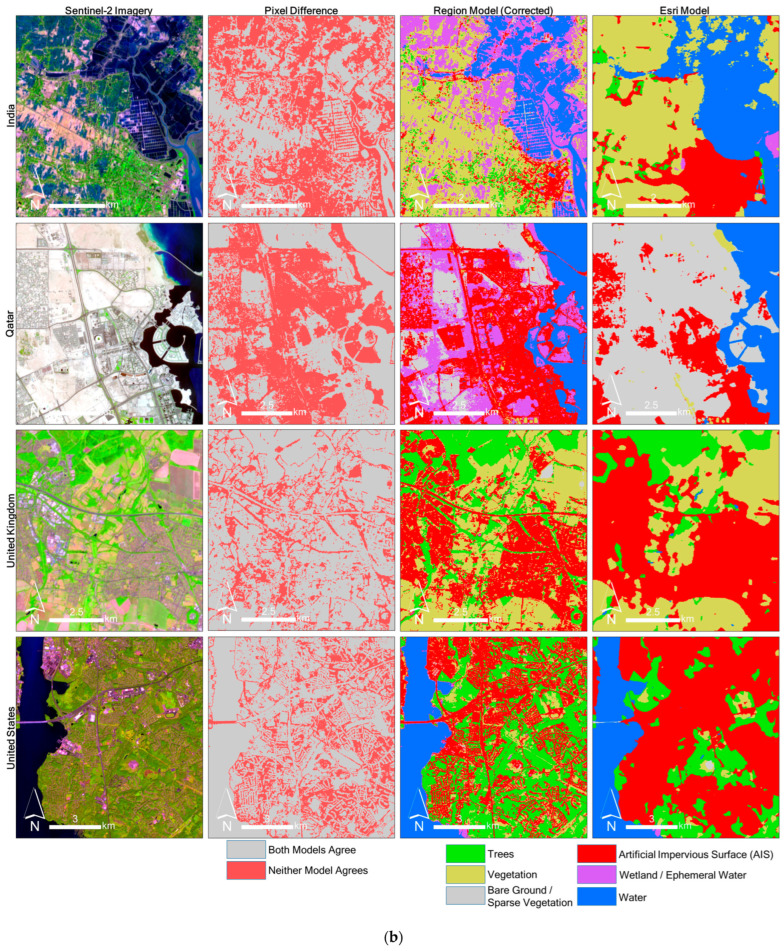
(**a**). Spatial variation between the Esri model and the region-corrected model for the BR, EG. FR sites (**b**) Spatial variation between the Esri model and the region-corrected model for the IN, US, UK sites.

Some of the previously mentioned omission or commission errors can be observed in Figure 10, where the region-corrected model and Esri model are shown zoomed to areas of notable variation within each study area. Within all scenes except QA, the AIS area appears to be overestimated in the Esri model. Many of the pixels surrounding true AIS areas are incorrectly classified as AIS. If this were a land use classification, then the Esri model would likely have very high AIS accuracy; however, for land cover, it is less accurate than our model in these locations. The Esri model is likely to have fewer AIS commission errors in agricultural areas (e.g., as seen in the U.K.) but more AIS omission errors from roadways and infrastructure outside of towns (e.g., as seen clearly in BR and EG scenes). Another interesting trend is observed in EG, where the Esri model maps swaths of pixels as vegetation, whereas the region-corrected model maps them as bare ground. Most of these pixels have very low NDVI values, typically lower than 0.20, but some do appear to be sparsely vegetated. This variation is likely due to a difference in definitions: bare ground/sparsely vegetated are grouped in one category for our region-corrected model, whereas the Esri model appears to assign sparsely vegetated pixels to the vegetation category. Another interesting model disagreement is seen in the IN scene. An area of seasonally inundated rice fields within the North 24 Parganas district is mapped by the Esri model as vegetation and by the region-corrected model as wetlands (depending on definitions, either could be considered correct). Another large area of seasonally inundated aquaculture is mapped by the Esri model as water and by the region-corrected model as wetlands.

**Figure 11:**
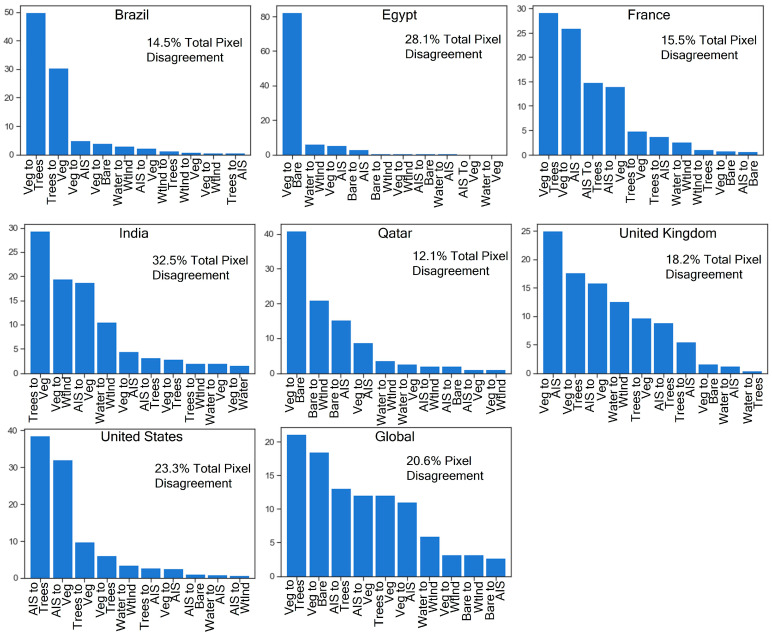
Pixel disagreement between the region-corrected model and Esri model for the entirety of each scene and across all seven scenes (global) for the six-class land cover scheme. The Esri model is the original condition, and the region-corrected model is the second condition (e.g., “AIS to Trees” indicates AIS in the Esri model, but Trees in the region-corrected model).

While the visualizations of spatial variation between the Esri model and the region-corrected model provide illustrative examples, they do not represent the entire study area. Figure 11 summarizes the variation between the two models and includes the top 10 most common variations in land cover between them. Overall, the three locations with the most pixel disagreement were IN at 32.5%, EG at 28.1%, and U.S. at 23.3%. Across all seven sites, the three most common land cover class pixel differences for the six-class land cover output were (1) vegetation (Esri), trees (region corrected), (2) vegetation (Esri), bare (region corrected), and (3) AIS (Esri), trees (region corrected). Vegetation-bare made up more than 80% of the pixel disagreement in EG and 40% in QA while occupying a drastically smaller proportion of disagreement in non-arid regions. Vegetation-AIS had the highest proportion of pixel disagreement for the U.K. scene at 25%; some of this disagreement is attributed to AIS commission errors in the region-corrected model, along with AIS rural road pixels omitted by the Esri model. Vegetation-trees and trees-vegetation together occupied nearly 80% of pixel disagreement in the BR scene, which contained many mixed pixels, young forest, and agricultural areas that posed a challenge to both models.

### 4.7. Areal Variation between the Different Land Cover Models

The corrections made to the regional and global land cover models resulted in improvements to the overall accuracy as previously described. The most impactful correction across all scenes was in EG, with the use of the 55 × 55 pixel window returning the maximum NDVI_95th_percentile and correcting AIS commission errors on bare ground when the NDVI_95th_percentile did not exceed 0.3 in that pixel window. The changes for EG were drastic (as seen earlier in Figure 4). For the global six-class model, the AIS area dropped from 9.8% to 5.1% with this correction, whereas in the regional six-class model, the impact was smaller, with a decrease from 4.2% to 2.8% due to the removal of large swaths of false positive AIS area occurring on bare ground. 

**Figure 12:**
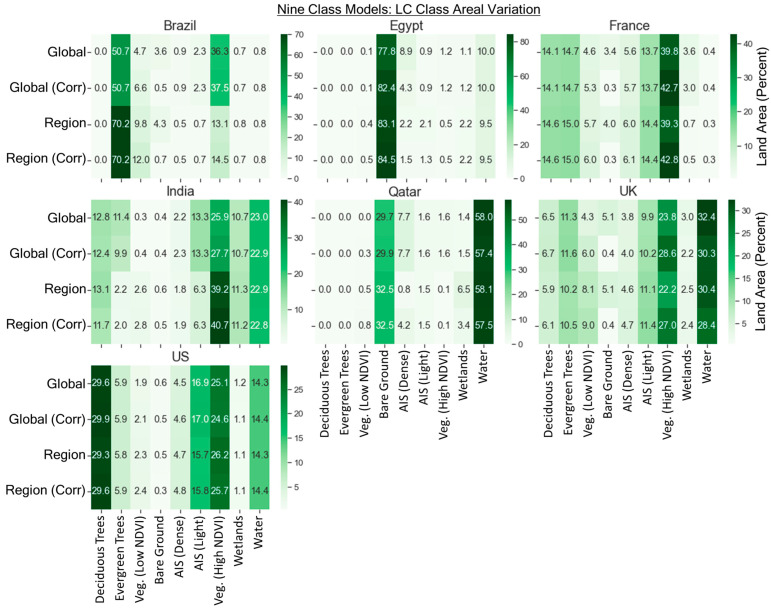
Percentage of areal variation per scene for each land cover type for the nine-class scheme.

**Figure 13:**
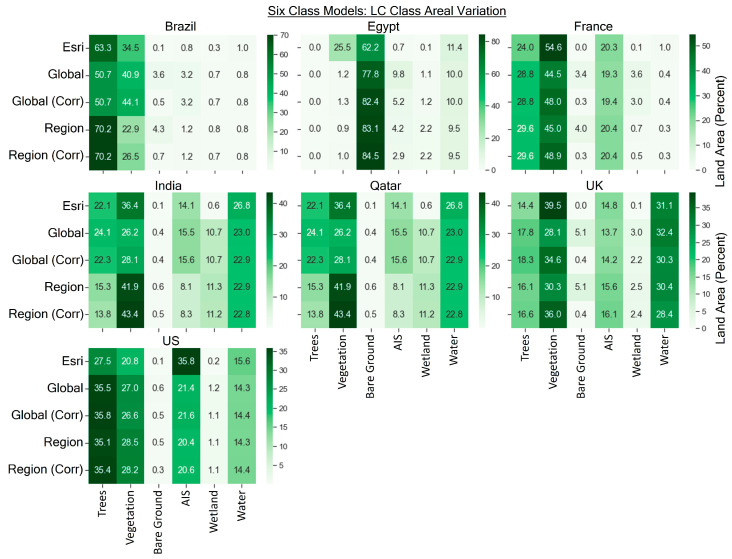
Percentage of areal variation per scene for each land cover type for the six-class scheme.

The areal variation in land cover classes for the different models is shown in Figure 12 for the nine-class models and Figure 13 for the six-class models. For the six-class model, we can see that corrections result in changes to many of the land cover classes, with the highest changes typically in the vegetation and bare ground classes, but also the AIS, water, and trees categories to a lesser extent. Two key differences are as follows: (1) U.K. bare ground decreases from 5.1% to 0.4% in the global and regional models (likely due to fallow agricultural fields that eventually become vegetated), and (2) vegetation varies in the EG scene between Esri and the global and regional models (25.5% vs. less than 1.5%). Much of this vegetated area predicted by Esri is detected as bare ground in the global and regional models from this study.

## 5. Discussion

The land cover maps from this study generally resulted in similar accuracies to those produced by other studies that leveraged more intensive time series of data or deep learning approaches. Considering this, our study’s novelty is that it offers a unique, less data-intensive way to generate accurate land cover maps without manual data labeling, usage of a novel NDVI texture metric, region- and global-based models, and a temporal correction, which have not been found in prior studies. In addition to the framework, a main contribution from this study is that it not only demonstrated that two dates of imagery can be used to create an accurate annual land cover product but that temporal corrections using NDVI 5th and 95th percentile composites can yield about 5% higher accuracy. The most drastic improvement with the temporal corrections was the conversion of false positive AIS to bare ground in EG, where the NDVI 95th percentile combined with a pixel neighborhood reduced these errors by about 33% in the region-corrected model.

This framework enables the on-demand generation of land cover types across international study sites. The temporally corrected region-based models generally yielded higher accuracy than the global model, but not always. The evaluation was carried out across seven geographically diverse sites using a robust and balanced accuracy assessment scheme encompassing the entirety of the Sentinel-2 granule (except the no-data areas in the BR scene and the QA and U.K. scene where easy-to-classify ocean areas beyond 1 km distance from the shoreline were excluded). In comparison to the Esri model, our study’s region-corrected model outperformed it in the six-class scheme (82.8% (±3.1%) vs. 72.8% (±3.5%). However, under the five-class scheme, we could not definitively say our model was more accurate with a 95% confidence interval (80.0% (±3.4%) Esri vs. 85.1% (±2.9%) region-corrected model). It should be noted that the Esri model had its own accuracy assessment, which reported about 85% accuracy for their nine-class model, which exceeds the accuracy of our nine-class model (73.1%). However, direct comparison was not possible due to class definition differences. The 10 m Dynamic World land cover dataset with nine land cover classes produced globally had F1 scores ranging from 77% to 88% based on different assessment schemes. Their approach was more accurate than our model, 73.1% vs. 77–88% [33]. However, their approach also used fully convolutional neural networks and cloud computing, with a large volume of time series imagery, compared to our less data- and compute-intensive approach. One study that used shallow machine learning methods and semi-automated training data labels to produce a thirteen-class land cover map yielded 86% overall accuracy; however, this study was geographically limited to Europe [28]. Further comparison against a global 2019 12-class Landsat-based classifier found similar, but slightly higher, accuracy levels to our study (78.3% vs. 73.1%) [90].

There are several important limitations to mention in this study. While we used robust accuracy assessments and sampling as guided by the literature [106], we were only able to evaluate seven different international sites. While diverse, this evaluation set did not include high-latitude locations or extremely mountainous terrain. For the ancillary data layers, there are several options available for AIS or forest cover that could likely be interchanged, but this study was more interested in developing a framework for automated classification, and evaluating different ancillary layers was beyond the scope of this study. Furthermore, the ancillary layers (forest cover, AIS, and SCL water) that were used (in modified form) as training data may contain their own data errors (e.g., false positive AIS), which can be carried over into the output produced from this study (as was very evident in the QA and EG scenes). The GISA dataset and forest cover dataset were both produced from 30 m Landsat imagery, which has different pixel alignment and size than the 20 m Sentinel-2 used in this study. While effort was taken to resample and align, this discrepancy could have led to some errors; however, these should be considered in the context of our accuracy assessment results. For our stepwise threshold evaluation on the binary training masks, we did not test all possible combinations due to computational needs and time constraints, but we demonstrated that accuracy typically did not vary drastically. It is also worth mentioning that the GISA layer was originally from 2016, which is now outdated. However, considering that AIS areas almost never decrease, this is acceptable to use for the selection of training data. These topics would be beyond the scope of the article but would be of interest to explore in future research.

The analysis indicated that the Esri land cover layer may define some land cover classes differently than our study. For example, their model seems to include sparsely vegetated pixels in the vegetation categories, whereas this study included them with bare ground. Furthermore, the Esri model appeared to consider any pixel that had water at any point during the year as water, whereas we considered such a pixel to be wetland/ephemeral water. Neither is necessarily more correct than the other, but this leads to differences that are not easily rectifiable in this study. The accuracy levels reported for the Esri model, therefore, may appear lower than reported in their original studies because we evaluated the Esri model against our ground truth and our own definitions of each land cover type. The purpose of the five-class and six-class model comparisons was to close the gap in land cover definitions between the two studies.

While our study evaluated the framework across seven international heterogeneous sites, future expansion to include evaluation across a continental or global scale would yield significant insights. However, this would require integration with cloud computing frameworks due to the high data storage and computational requirements, as well as more extensive accuracy assessment. We expect that the workflow would need further refinements in extremely mountainous and arctic regions where shadows and terrain drastically impact spectral signatures [110]. However, some efforts have been made to overcome these issues in various land cover mapping applications using advanced segmentation and convolutional neural networks [111]. However, considering these shadow correction advancements, it is currently not possible to obtain spectral values when a pixel is completely occluded (i.e., pixel value = 0), but adjustments can be made when values are nonzero [112].

## 6. Conclusions

This study developed an approach to accurately map land cover types using two dates of Sentinel-2 20 m imagery and ancillary layers with automatically generated training data for subsequent classification using a random forest. This framework used logical decision functions based on Euclidean distances, texture, and spectral index values to create binary land cover masks representative of central/ideal pixel conditions that were then used for sampling spectrally balanced training data and subsequent classification with a random forest. A total of 2858 stratified random accuracy points spread across the seven geographically diverse study sites were generated on this intermediate set of land cover maps and then used for stepwise threshold optimization of the binary land cover training masks. The thresholds were optimized globally (across all seven scenes) and regionally (arid, temperate, and tropics). Temporal corrections, including a novel texture filter to reduce confusion between AIS and bare ground, were applied post-classification using annual composites of the 95th and 5th percentile of NDVI, which resulted in an average of 5% increase in accuracy for the region models and a 3% average increase for the global models (e.g., nine-class region model 68.4% vs. 73.1%). The region model was slightly less accurate than the global model; however, the region-corrected models were more accurate than the global-corrected models (except for nine-class). The six-class and five-class land cover schemes had accuracies for the region-corrected models of 82.8% (±3.1%) and 85.1% (±2.9%), respectively. A comparison of these two models against a deep-learning-based model from Esri found that the Esri models performed slightly less accurately at 72.8% (±3.5%) and 80.0% (±3.4%) for six-class and five-class schemes, respectively. Ultimately, this study devised a methodology to automatically map land cover type accurately with two dates of Sentinel-2 imagery and shallow machine learning models without a compute-intensive deep-learning- or dense time series-based approach. Future work could explore the optimization of this workflow in challenging mountainous terrain and refinements as new ancillary layers are released. Ultimately, as compute capabilities increase, conversion of the approach toward a deep learning classifier could yield improvement.

## Figures and Tables

**Table 1:** Study area and imagery details for the seven sites.

Site Name	Sentinel-2 Granule ID	Imagery Date (Winter)	Imagery Date (Summer)	Region	Geographical Features	Prevalent Land Covers	Largest City Population
Maranhão, Brazil (BR)	T23MQR	2019/DEC/21	2020/AUG/07	Tropics	Tropical forest, riverine	Forest, agriculture	Coelho Neto (~50K)
Aswan, Egypt (EG)	T36QVM	2021/DEC/26	2021/AUG/18	Arid	Lake, hilly terrain	Bare ground, water	Aswan (~380K)
Paris, France (FR)	T31UDQ	2020/NOV/26	2021/JUN/14	Temperate	Mosaic landscape	Built-up, agriculture	Paris (~11 million)
Kolkata, India (IN)	T45QXE	2018/FEB/04	2018/OCT/22	Tropics	Mega urban, aquaculture, rice, mangrove	Built-up, Agriculture, Wetlands	Kolkata Metro Area (~15 million)
Doha, Qatar (QA)	T39RWJ	2022/MAR/10	2022/SEP/11	Arid	Coastal, bare ground	Bare Ground, Built-up	Doha (~2.3 million)
Portsmouth, United Kingdom (UK)	T30UXB	2019/NOV/18	2020/JUL/30	Temperate	Mosaic landscape	Agriculture, Built-up	Southampton ~(900K)
Washington, DC, USA (US)	T18SUJ	2021/SEP/02	2021/DEC/26	Temperate	Suburban sprawl	Built-up, Forest	DC (~700K)

**Table 2:** Names of the land cover classes used in this study.

Nine Class Land Cover	Six Class Land Cover	Five Class Land Cover
Deciduous Trees	Trees	Trees
Evergreen Trees	Vegetation	Vegetation
Vegetation (Low NDVI)	Bare Ground/Sparse Vegetation	Bare Ground/Sparse Vegetation
Vegetation (High NDVI)	AIS	AIS
Bare Ground/Sparse Vegetation	Wetland/Ephemeral Water	Wetland/Ephemeral Water/Water
Artificial Impervious Surface (AIS) High Density	Water	
AIS Low Density		
Wetland/Ephemeral Water		
Water		

**Table 3:** Data layers used to create the binary land cover masks used for training data sampling.

Mask	Global Tree Cover Layer	Global AIS Layer	Mid-Infrared (MIR) Reflectance	NDVI	NDVI Texture	NDWI	MNDWI	AWEI	Distance to SCL Water
AIS (Dense)		✓		✓	✓				✓
AIS (Light)		✓		✓	✓				✓
Water		✓ **(distance)**	✓			✓	✓	✓	
Temporary Water/Wetland		✓ **(distance)**	✓			✓	✓	✓	
Deciduous Trees	✓	✓ **(distance)**		✓			✓		✓
Evergreen Trees	✓	✓ **(distance)**							
Vegetation (Low NDVI)	✓ **(distance)**	✓ **(distance)**	✓	✓					
Vegetation (High NDVI)	✓ **(distance)**	✓ **(distance)**		✓					
Bare Ground/Sparse Vegetation		✓ **(distance)**	✓	✓					✓
Snow		*Internal Sentinel-2 Snow Mask from SCL*							

**Table 4:** The most accurate threshold values for each of the binary land cover training masks as determined by the stepwise threshold analysis in comparison to the ground truth points.

Land Cover Training Mask	Variable	Most Accurate Value (Arid)	Most Accurate Value (Temperate)	Most Accurate Value (Tropics)	Most Accurate Value (Global)
Vegetation (High NDVI)	Minimum NDVI	0.5	0.1	0.1	0.1
	Minimum GISA AIS Distance	200 m	200 m	100 m	100 m
Bare Ground/Sparse Vegetation	Minimum GISA AIS Distance	300 m, 600 m (Tie)	300 m	300 m	300 m
	Minimum Band 11 reflectance	0	600	1200	1200
Wetland/Ephemeral Water	Minimum NDVI	0.0	0.1	0.1	0.1
	Minimum MNDWI	−0.25	−0.15, −0.20 (Tie)	−0.25	−0.20
	Minimum GISA AIS Distance	200 m	None	None	None
Water	Minimum Spectral Index Values (MNDWI, NDWI, AWEI)	−0.05	−0.03	−0.05	−0.03
	Maximum Band 11 Reflectance	1500	None	1500	1500
Vegetation (Low NDVI)	Minimum NDVI	0.3	0.4	0.3	0.3
	Maximum NDVI	0.5	0.5	0.6	0.5
	Minimum Band 11 reflectance	600	600	1200	1200
AIS (Low Density and High Density)	Natural Breaks (Jenks)	Tie	Natural Breaks = True	Tie	
Deciduous and Evergreen Trees	Natural Breaks (Jenks)	Tie	Tie	Tie	
Vegetation (Low NDVI and High NDVI)	Natural Breaks (Jenks)	Tie	Tie	Natural Breaks = False	

**Table 5:** Confusion matrixes of the six-class and five-class land cover schemes of the region-corrected model (bottom) and the Esri model (top). F1 score, overall, producer’s, and user’s accuracies are shown.

**6 Class Esri**		REFERENCE								**5 Class Esri**		REFERENCE					
**Class**	**Trees**	**Vegetation**	**Bare Ground**	**AIS**	**Wetland**	**Water**	**Total**	**User’s Acc.**		**Class**	**Trees**	**Vegetation**	**Bare Ground**	**AIS**	**Water/Wetland**	**Total**	**User’s Acc.**
**Trees**	321	72	1	12	26	1	433	74.1%		**Trees**	321	72	1	12	27	433	74.1%
**Vegetation**	32	624	133	33	61	3	886	70.4%		**Vegetation**	32	624	133	33	64	886	70.4%
**Bare Ground**	0	5	266	15	7	0	293	90.8%		**Bare Ground**	0	5	266	15	7	293	90.8%
**AIS**	59	54	16	494	9	4	636	77.7%		**AIS**	59	54	16	494	13	636	77.7%
**Wetland**	2	5	0	0	20	3	30	66.7%		**Water/Wetland**	3	11	10	5	581	610	95.2%
**Water**	1	6	10	5	202	356	580	61.4%		**Total**	415	766	426	559	692		
**Total**	415	766	426	559	325	367				**Producer’s Acc.**	77.3%	81.5%	62.4%	88.4%	84.0%		
**Producer’s Acc.**	77.3%	81.5%	62.4%	88.4%	6.2%	97.0%											
																**Overall Acc.**	80.0% (±3.4%)
							**Overall Acc.**	72.8% (±3.5%)								**Producer’s Acc.**	78.7%
							**Prod. Acc**	68.8%								**User’s Acc.**	81.7%
							**User’s Acc.**	73.5%								**F1**	0.802
							**F1**	0.711									
**6 Class Region Corr.**		REFERENCE								**5 Class Region Corr.**		REFERENCE					
**Class**	**Trees**	**Vegetation**	**Bare Ground**	**AIS**	**Wetland**	**Water**	**Total**	**User’s Acc.**		**Class**	**Trees**	**Vegetation**	**Bare Ground**	**AIS**	**Water/Wetland**	**Total**	**User’s Acc.**
**Trees**	363	57	0	2	23	0	445	81.6%		**Trees**	363	57	0	2	23	445	81.6%
**Vegetation**	47	632	14	39	24	3	759	83.3%		**Vegetation**	47	632	14	39	27	759	83.3%
**Bare Ground**	0	1	353	33	2	0	389	90.7%		**Bare Ground**	0	1	353	33	2	389	90.7%
**AIS**	5	69	50	469	22	2	617	76.0%		**AIS**	5	69	50	469	24	617	76.0%
**Wetland**	0	2	14	12	221	34	283	78.1%		**Water/Wetland**	0	2	14	16	616	648	95.1%
**Water**	0	0	0	4	33	328	365	89.9%		**Total**	415	761	431	559	692	2858	
**Total**	415	761	431	559	325	367				**Producer’s Acc.**	87.5%	83.0%	81.9%	83.9%	89.0%		
**Producer’s Acc.**	87.5%	83.0%	81.9%	83.9%	68.0%	89.4%											
																**Overall Acc.**	85.1% (±2.9%)
							**Overall Acc.**	82.8% (±3.1%)								**Producer’s Acc.**	85.1%
							**Producer’s Acc.**	82.3%								**User’s Acc.**	85.3%
							**User’s Acc.**	83.3%								**F1**	0.852
							**F1**	0.828									

**Table 6:** Confusion matrixes of the nine-class global- and region-corrected models produced from this study.

**9 Class Global corr.**		REFERENCE									
**Class**	**Deciduous Trees**	**Evergreen Trees**	**Veg (Low)**	**Bare Ground**	**AIS (High)**	**AIS (Low)**	**Veg (High)**	**Wetland**	**Water**	**Total**	**User’s Acc.**
**Deciduous Trees**	128	19	3	0	1	3	41	13	0	208	61.54%
**Evergreen Trees**	48	167	0	0	0	0	17	16	0	248	67.34%
**Veg (Low)**	0	0	65	7	4	8	12	4	1	101	64.36%
**Bare Ground**	0	0	0	342	18	2	0	2	0	364	93.96%
**AIS (High)**	0	0	6	56	251	46	1	14	0	374	67.11%
**AIS (Low)**	8	2	68	9	61	152	37	17	3	357	42.58%
**Veg (High)**	15	28	57	14	2	4	453	16	1	590	76.78%
**Wetland**	0	0	0	3	4	2	1	217	39	266	81.58%
**Water**	0	0	0	0	1	0	0	26	323	350	92.29%
**Total**	199	216	199	431	342	217	562	325	367		
**Producer’s Acc.**	64.32%	77.31%	32.66%	79.35%	73.39%	70.05%	80.60%	66.77%	88.01%		
										**Overall Acc.**	73.4% (±1.8%)
										**Producer’s Acc.**	70.3%
										**User’s Acc.**	71.9%
										**F1**	0.688
**9 Class Region Corr.**		REFERENCE									
**Class**	**Deciduous Trees**	**Evergreen Trees**	**Veg (Low)**	**Bare Ground**	**AIS (High)**	**AIS (Low)**	**Veg (High)**	**Wetland**	**Water**	**Total**	**User’s Acc.**
**Deciduous Trees**	124	28	0	0	1	1	14	11	0	179	69.27%
**Evergreen Trees**	50	161	1	0	0	0	42	12	0	266	60.53%
**Veg (Low)**	4	4	127	8	5	24	47	5	2	226	56.19%
**Bare Ground**	0	0	1	353	24	9	0	2	0	389	90.75%
**AIS (High)**	0	0	6	37	231	49	1	9	0	333	69.37%
**AIS (Low)**	3	2	31	13	68	121	31	13	2	284	42.61%
**Veg (High)**	18	21	32	6	3	7	426	19	1	533	79.92%
**Wetland**	0	0	1	14	6	6	1	221	34	283	78.09%
**Water**	0	0	0	0	4	0	0	33	328	365	89.86%
**Total**	199	216	199	431	342	217	562	325	367		
**Producer’s Acc.**	62.31%	74.54%	63.82%	81.90%	67.54%	55.76%	75.80%	68.00%	89.37%		
										**Overall Acc.**	73.2% (±1.9%)
										**Producer’s Acc.**	71.0%
										**User’s Acc.**	70.7%
										**F1**	0.683

## Data Availability

All data used in this study were accessed from publicly available repositories. The Sentinel-2 imagery was provided by the European Space Agency (https://scihub.copernicus.eu/ accessed on various dates, 2022 and 2023). The global forest cover extent and loss layers were obtained from the University of Maryland, Global Land Analysis & Discovery (GLAD) Group (https://www.glad.umd.edu/dataset (accessed on 13 October 2022)). The GISA AIS layer was downloaded from https://zenodo.org/records/5791855 (accessed on 15 October 2022).
